# Could coagulase negative staphylococci be an evolutionary source of resistance genes for *Staphylococcus aureus*?

**DOI:** 10.1186/1753-6561-9-S1-A43

**Published:** 2015-01-14

**Authors:** P Jayadev-Menon, H Humphreys, D Fitzgerald-Hughes

**Affiliations:** 1 Royal College of Surgeons in Ireland, 123 St. Stephen’s Green, Dublin 2. Ireland; 2 RCSI Education and Research Centre, Beaumont Hospital, Dublin 9, Ireland

## Background

The remarkable ability of *Staphylococcus aureus* to acquire mobile genetic elements that carry virulence and antibiotic resistance genes, contributes to its success, pathogenicity and evolution. There is indirect evidence that methicillin-resistant *S. aureus* (MRSA) clones arose from genetic transfer of staphylococcal cassette chromosome mec (SCCmec) from Staphylococcus epidermidis and other coagulase-negative staphylococci (CNS) to methicillin-susceptible *S. aureus* (MSSA). These staphylococcal species are often co-located in human reservoirs such as the nose and this niche may provide an environment in which genetic transfer is favoured. The aim of this study was to demonstrate that methicillin-resistant coagulase-negative staphylococci (MRCNS) are commonly found in the nose of MRSA-positive patients and to provide evidence of antibiotic resistance gene transfer among these species.

## Methods

We developed a method for the recovery of MRCNS from MRSA-positive swabs. Colonies recovered from the nasal swab were identified as MRSA and MRCNS through phenotypic characteristics. Genetic analysis using multiplex PCR for *S. aureus* marker gene and methicillin resistance gene mec-A further confirmed the identity of these colonies. Paired isolates of MRSA and MRCNS recovered from the same clinical site, were stored on cryoprotect beads at -80oC for further investigation.

## Results

MRCNS and MRSA were recovered from the same swab in approximately 45% of swabs investigated. Comparative DNA microarray analysis of five matched MRSA and MRCNS pairs indicated that they had mecA, and SCCmec elements in common suggesting that they share the same mec gene complex (SCCmecIV).

## Conclusions

MRCNS and MRSA share a common niche in the human nose and harbour similar antibiotic resistance genes that reflect a history of genetic transfer between these species. *S. epidermidis* and other CNS may serve as a potential reservoir for evolution of MRSA strains with enhanced virulence and antimicrobial resistance.

